# Effects of ectopic HER-2/neu gene expression on the COX-2/PGE_2_/P450arom signaling pathway in endometrial carcinoma cells: HER-2/neu gene expression in endometrial carcinoma cells

**DOI:** 10.1186/1756-9966-32-11

**Published:** 2013-03-02

**Authors:** Shu Li, XiaoXin Ma, Li Ma, Cuicui Wang, YuanQi He, ZhiJuan Yu

**Affiliations:** 1 Department of Gynecology and Obstetrics, Shengjing Hospital of China Medical University, Shenyang, 110004, China

**Keywords:** HER-2/neu, COX-2, P450arom, Estradiol, Endometrial carcinoma cell

## Abstract

**Objectives:**

To investigate the role of HER-2/neu-mediated COX-2/P450arom signal in estrogen-dependent endometrial carcinoma.

**Methods:**

The recombinant eukaryotic expression vector, pcDNA3.1-HER-2/neu, was constructed and transfect to Ishikawa endometrial carcinoma cells. The expression of COX-2 and P450arom in transfected cells were detected by real-time PCR and western blotting. The levels of estrogen in cell supernatants were detected by ELISA.

**Results:**

Over-expression of HER-2/neu in transfected cells was confirmed by real-time PCR and western blotting. The levels of autocrine estrogen in transfected cells was significantly increased which combination with the enhancement of COX-2 and P450arom expression in transfected cells.

**Conclusion:**

HER-2/neu induced the improvement of autocrine estrogen in endometrial carcinoma cell through triggering the COX-2/P450arom signal.

## Introduction

Endometrial carcinoma is a common gynecologic malignancy with uncharacterized molecular mechanisms of pathogenesis. A large body of studies has reported that the origin of endometrial carcinoma was associated with long-term estrogen stimulation without counteraction [1]. Long-term stimulation of estrogen can cause endometrial hyperplasia, even atypical hyperplasia, and can progress to carcinogenesis. Local synthesis of estrogen may also lead to endometrial carcinoma. A better understanding of the mechanisms of local estrogen synthesis is important to find the new treatment of endometrial carcinoma.

 Aromatase (P450arom), a key enzyme for the bio-synthesis of estrogens, was detectably active and over-expressed in endometrial carcinoma, whereas it was undetectable in normal endometria. P450arom is the rate-limiting enzyme that catalyzes the final step in the conversion pathway from androgen to estrogen. The quantity and activity of P450arom can directly affect the levels of estrogen in normal or abnormal tissues, in order to maintain estrogen-related physiologic functions in normal tissues. Meanwhile, P450arom play a role in the pathogenesis and prognosis of estrogen-dependent diseases. The activity of P450arom is regulated by prostaglandin E2 (PGE_2_), which is affected by cyclooxygenase-2 (COX-2). We hypothesize that COX-2/PGE_2_/P450arom might be a signaling pathway in estrogen-dependent diseases to regulate the autocrine activity of estrogen in cancerous tissues. Previous reports indicated that HER-2/neu regulated the expression of COX-2 as the upstream molecular of COX-2-mediated signal pathways [2,3]. In the present paper, our results demonstrated that transfection with HER-2/neu in endometrial cells induced the activation of COX-2/PGE_2_/P450arom signal, resulting in the increase of autocrine estrogen from endometrial cells.

## Materials and methods

### Cell culture

The Ishikawa cell line was kindly supplied by the Department of Pathophysiology, Beijing University. Cells were cultured in RIPM1640 with 10% fetal bovine serum, 100 U/ml penicillin, and 100 μg/ml streptomycin in an incubator maintained at 37°C and 5% CO_2_. Celecoxib, a selective COX-2 inhibitor, was purchased from Santa Cruz Biotechnology and dissolved in DMSO to generate a 100 mM stock solution that was stored at −20°C. For inhibition experiment, confluence cells were starved by serum deprivation overnight. Then, cells were treated with 80 μM celecoxib and incubated for 48 h.

### Construction of pcDNA3.1-HER2/neu

Upstream (5^′^-TGGGAGCCTGGCATTTCTG-3^′^) and downstream (5^′^-TCCGGCC ATGCTGAGATGTA-3^′^) primers were designed based on *HER-2/neu* cDNA sequence obtained from GenBank. For cloning, HindIII/XbaI restriction endonuclease sites were inserted flanking the target gene primers. Primers were synthesized by TaKaRa Biotechnology Co., Ltd. Total RNA was isolated from Ishikawa cells using TRIzol reagent (TaKaRa, China) according to the manufacturer’s instructions. *HER-2/neu* cDNA was reverse-transcribed using the One Step RNA PCR Kit (TaKaRa) according to the manufacturer’s recommendations. PCR conditions included denaturation at 94°C for 5 min, 25 cycles of denaturation at 94°C for 45 s, annealing at 60°C for 1 min, and extension at 72°C for 6 min, with a final extension at 72°C for 10 min. PCR products were separated on 1% agarose gel and eluted. The PCR product was sent to TaKaRa for sequencing. PcDNA3.1 plasmid and *HER2* cDNA were digested with HindIII/XbaI double endonucleases. The digested products were separated by agarose gel electrophoresis and purified. Pure *HER2* cDNA and vector were mixed at a 4:1 ratio and were ligated at 16°C for 20 h. Ligation products were transformed into *E. coli*. After culturing at 37°C overnight, *E. coli* cells were screened. DNA was isolated from positive colonies (named pcDNA3.1 (+)-HER2), and were sequence-verified.

### Gene transfection and G418 screening

pcDNA3.1 (+)-HER2 was isolated using a plasmid extraction kit (Invitrogen, USA) according to the manufacturer’s protocol. Ishikawa cells in logarithmic growth were transferred to 6-well culture plates at 1 × 10^6^ cells/well and were cultured for 1 d prior to transfection. Ishikawa cells then were transfected with pcDNA3.1-HER-2/neu or pcDNA3.1 (control) using Lipofectamine2000 (Invitrogen, USA) according to the manufacturer’s protocol. Non-transfected cells were cultured as the negative control. The original culture media was discarded 3 d after transfection, and cells were cultured in complete media containing 10% fetal bovine serum and 700 μg/ml of G418 (Invitrogen, USA). G418-resistant clones were selected and transferred to culture media containing 350 μg/ml of G418 for scale-up culture.

### RNA interference

To knock down the HER2/neu in Ishikawa cells, the siRNA transient transfection experiment was performed according to the previous publication [4]. Briefly, Ishikawa cells were transfected with 25 nM COX-2 siRNAs, respectively. Non-targeting siRNA was used as negative control. Transfections were carried out according to the guidelines for the DharmaFECT® siRNA Transfection Reagents (Dharmacon). Ishikawa cells were collected at 72 hours post-siRNA addition for protein western blotting analysis.

### Real-time RT-PCR analysis of *HER-2/neu*

Total RNA was extracted from Ishikawa cells stably transfected with pcDNA3.1 (+)-HER2 using TRIzol, as above. The same *HER-2/neu* primers were used as in the construction of pcDNA3.1 (+)-HER2. GAPDH was used as the internal control (upstream 5^′^-CATCCATGACAACTTTGGTATC-3^′^; downstream 5^′^-CCATCACGCCACAGTTTC-3^′^). cDNA was synthesized from 1 μg of total RNA using oligo(dT) primers in the presence of reverse transcriptase. Gene amplification was performed on a real-time PCR instrument (TaKaRa, China) using 1 μl of cDNA as template in a 25 μl volume. PCR was started at 95°C for 5 min, followed by 30 cycles of denaturation at 95°C for 10 s, annealing at 59°C for 15 s, and extension at 72°C for 20 s, with a final extension at 72°C for 10 min. Fluorescence intensity was monitored and recorded in real time. A melting curve analysis was performed after amplification was complete. The ΔΔCt value was used to evaluate expression levels of HER2 and COX-2 mRNA. By this method, a higher expression level is related to a lower ΔΔCt value.

### Western blotting for HER-2/neu, COX-2, and P450arom

Cells collected from non-transfected, pcDNA3.1-transfected, and pcDNA3.1-HER2-transfected groups were lysed with 250 μl protein extracting fluid (RIPA lysis buffer: 50 mM Tris [pH 7.4], 150 mM NaCl, 1% Triton X-100, 1% sodium deoxycholate, 0.1% SDS), homogenized for 10 min, incubated in an ice-bath for 1 h, and centrifuged at 12,000 × *g* for 30 min at 4°C. Supernatants were collected, and protein concentrations were determined using the BCA protein assay system (Pierce, USA). Proteins were separated by 12% SDS-PAGE and were transferred to PVDF membranes. After blocking overnight at 4°C in 1 × PBS, 0.1% Tween 20, and 5% non-fat milk, membranes were incubated with anti-HER-2/neu (1:800), COX-2 (1:400), P450arom (1:400) and β-actin (1:800) polyclonal antibodies (Santa Cruz Biotechnology, USA) for 3 h at room temperature. Membranes were washed twice and incubated with horseradish peroxidase-conjugated goat anti-rabbit secondary antibody (ZhongShan, China, 1:1,500) for 2 h at room temperature. Immunodetection was performed by chemiluminescence (ECL reagent, Beyotime, China) and membranes were exposed to film. Images were captured using a transmission scanner. For quantification, target proteins were normalized to β-actin (the internal standard) by comparing the gray-scale values of proteins to corresponding β-actin values. Quantification was performed using UVP Gelworks ID Advanced v2.5 software (Bio-Rad, USA).

### ELISA for PGE_2_ and E_2_ detection

Supernatants were collected from non-transfected, pcDNA3.1-transfected, and pcDNA3.1-HER2-transfected groups for ELISA. Supernatant PGE_2_ and E_2_ concentrations were measured using an ELISA kit (R&D Systems, Minneapolis, MN, USA) according to the manufacturer’s instructions. Each sample was examined in triple and averaged for data analysis.

### Statistical methods

SPSS v10.0 software was used for all statistical analyses. Data were expressed as mean ± standard error of the mean (SEM). One-factor analysis of variance was used for pairwise comparison. Statistical significance was defined as *P* < 0.05.

## Results

### Construction of pcDNA3.1-HER2

**Figure 1:**
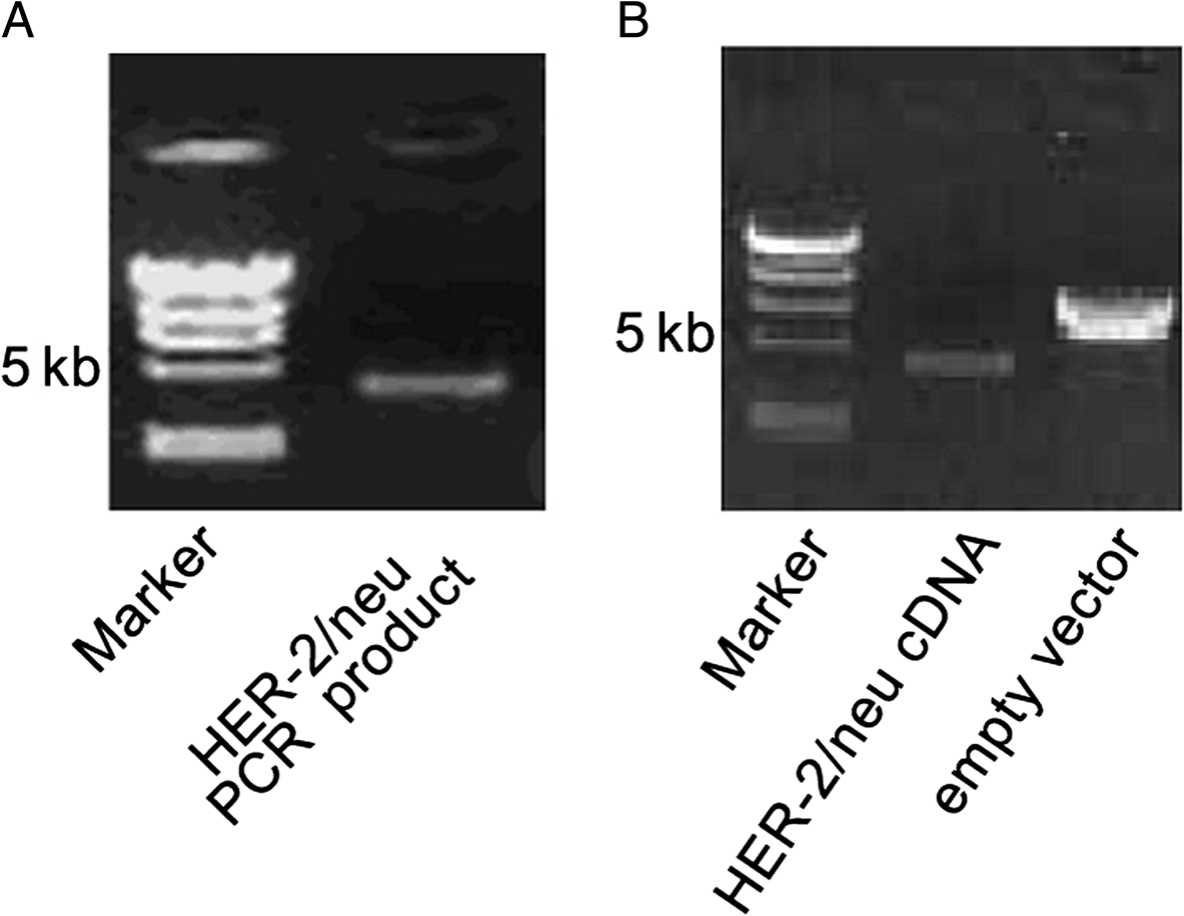
**RT-PCR and digestion products. A**. HER-2/neu RT-PCR, Marker: λ-HindIII DNA marker; **B**. Digestion. Markker: λ-HindIII DNA marker.

RT-PCR of *HER-2/neu* yielded a specific band of approximately 4.4 kb (Figure 1A). The DNA fragment sizes from *HER-2/neu* cDNA and pcDNA3.1 plasmid digested with HindIII and XbaI were as predicted from the sequence (Figure 1B). DNA sequencing confirmed the absence of point or frameshift mutations in HER-2/neu cDNA.

### Expression of HER-2/neu in Ishikawa cells stably transfected with pcDNA3.1-HER2

**Figure 2:**
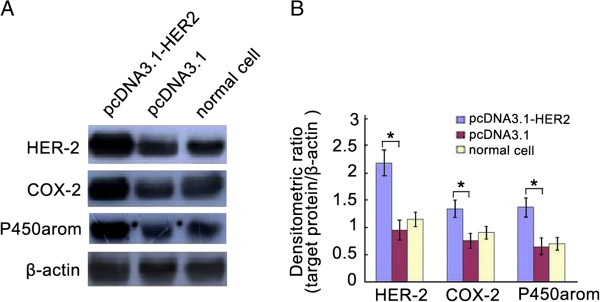
**The levels of HER-2/neu, COX-2, and P450armo in over-expressed HER2 ishikawa cells were detected by western blotting. A**. Represent image for western blot. **B**. Analysis of protein levels in each group and quantification of band density was done using Image J. * *P* < 0.05.

Real-time RT-PCR demonstrated significantly higher HER-2/neu mRNA expression in pcDNA3.1-HER2-transfected cells compared with empty plasmid-transfected or non-transfected cells (Table 1). Western blotting indicated a significant increase in HER-2/neu protein levels of cells transfected with pcDNA3.1-HER2 compared with empty plasmid-transfected or non-transfected cells (Figure 2). These results imply that the transfection was effective, and that the cells were appropriate for subsequent analyses.

**Table 1:** HER-2/neu mRNA expression

**Group**	**ΔCt**	**-ΔΔCt**	**2**^**-ΔΔCt**^
**HER-2 transfected**	**97.16 ± 0.71**	**2.62 ± 0.71**	6.15 (3.75–10.06)*
**pcDNA3.1 transfected**	**9.88 ± 1.10**	**0.1 ± 0.10**	**1.07 (1.06–1.08)**
**Non-transfected**	**9.78 ± 1.09**	**0 ± 1.09**	**1 (0.47-2.13)**

* *P* < 0.05.

### Transfected with pcDNA3.1-HER2 in Ishikawa cells induced the increase of COX-2, PGE_2_ and P450arom expression

Western blotting demonstrated that levels of COX-2 and P450arom in Ishikawa cells stably transfected with pcDNA3.1-HER2 were significantly higher compared to those in empty plasmid-transfected or non-transfected cells (Figure 2). In additionally, ELISA analysis showed that the supernatant level of PEG_2_ in pcDNA3.1-HER2-transfected group was significant higher than that of the empty plasmid-transfected group, and the normal cell group.

### Transfected with pcDNA3.1-HER2 induced the increase of autocrine E_2_ from Ishikawa cells

ELISA indicated was there were statistically significant differences in the cell supernatants of E_2_ levels among the pcDNA3.1-HER2-transfected group, the empty plasmid-transfected group, and the normal cell group (Table 2).

**Table 2:** **ELISA analyses for PGE**_**2 **_**and E**_**2 **_**in the supernatants of endometrial carcinoma cells**

**Group**	**PGE**_**2**_**(pg/ml)**	**E**_**2 **_**(pg/ml)**
Transfected	41.69 ± 0.87*	31.49 ± 2.14*
pcDNA3.1 transfected	31.35 ± 1.06	21.16 ± 2.37
Non-transfected	27.67 ± 1.20	20.56 ± 3.27

* *P* < 0.05.

### Inhibition of HER2 in Ishikawa cells induced the decrease of COX-2 and P450arom expression

**Figure 3:**
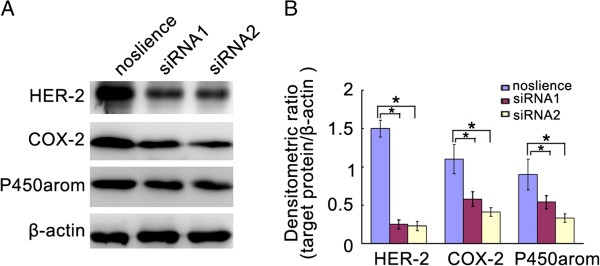
**The levels of COX-2, and P450armo in the ishikawa cells transfencted with HER2 siRNA. A**. Represent image for western blot. **B**. Analysis of protein levels in each group and quantification of band density was done using Image J. * *P* < 0.05.

RNA interference technology was used for the down-regulation of HER2 expression in Ishikawa cells. As shown in Figure 3, HER2 siRNAs were effectively able to knockdown the levels of HER2 in Ishikawa cells. Interestingly, down-regulation of HER2 expression induced significantly the reduction of COX-2 and P450arom levels in Ishikawa cells (Figure 3).

### Inhibition of COX-2 in the over-expressed HER2 Ishikawa cells led to the decrease of PGE2 and P450arom expression

**Figure 4:**
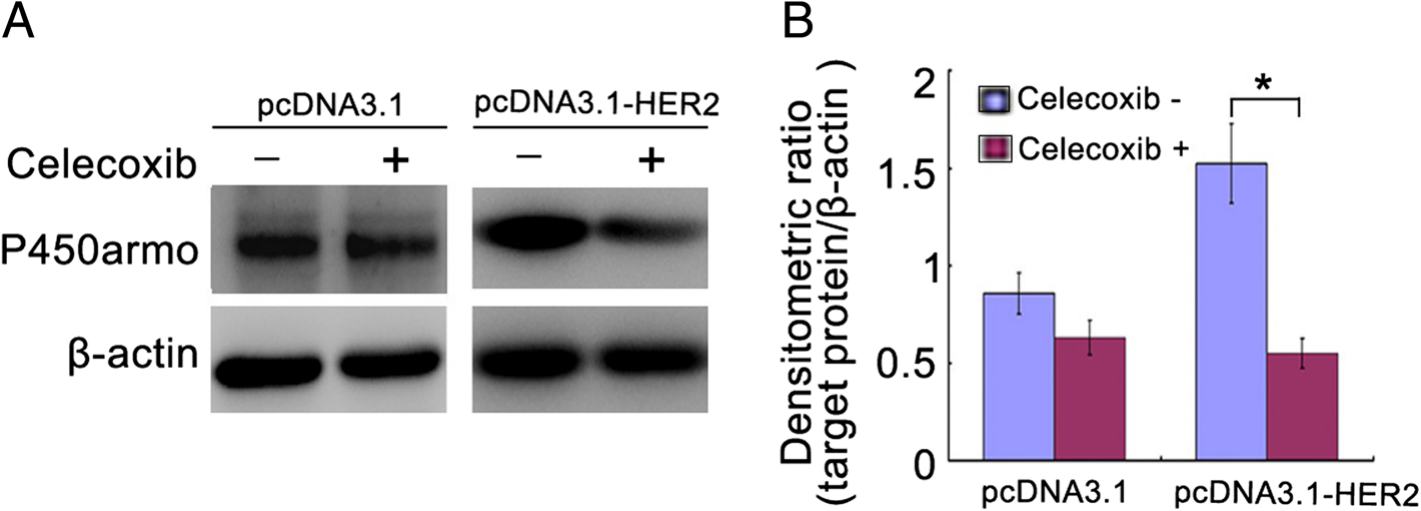
**The levels of P450armo in the ishikawa cells treated with 80 μM celecoxib. A**. Represent image for western blot. **B**. Analysis of protein levels in each group and quantification of band density was done using Image J. * *P* < 0.05.

To further investigate the relationship between the COX-2/PGE_2_/P450arom signal and HER2, celecoxib, a selective COX-2 inhibitor, was used for inhibition experiment. The results showed that inhibition of COX-2 in the over-expressed HER2 Ishikawa cells led to the obvious decrease of PGE2 and P450arom expression (Figure 4; Table 3).

**Table 3:** **ELISA analysis for PGE**_**2 **_**in the supernatants of tranfected endometrial carcinoma cells treated with Celecoxib**

**Group**	**Celecoxib - (pg/ml)**	**Celecoxib + (pg/ml)**
pcDNA3.1-HER-2	41.09 ± 2.76	33.86 ± 3.11*
pcDNA3.1	33.94 ± 3.41	30.56 ± 3.08

* *P* < 0.05.

## Discussion

An important member of the epidermal growth factor receptor (EGFR) family, the proto-oncogene HER-2/neu encodes a 185-kD transmembrane glycoprotein with tyrosine kinase activity [5]. HER-2/neu over-expression typically occurs in the placenta, embryonic epithelial tissue, and several types of tumor cells. In contrast, HER-2/neu is absent or minimally expressed in normal tissues [6]. The positive expression rate of the HER-2/neu protein in endometrial carcinoma is associated with clinical staging, a lower degree of tissue differentiation, and lymph node metastasis [7]. We have applied RT-PCR and ELISA to detect the expression of HER-2/neu, COX-2, p450arom and PGE2 in normal endometrium, hyperplasia endometrium and endometrial carcinoma respectively. The results showed that the expression of HER-2/neu was significantly correlated with pathologic grading, FIGO staging, and lymph node metastasis. But it has no correlation with menopausal status [8]. There are some studies also shows that the HER-2/neu gene contributes to the progression of carcinomas and tumor resistance to chemotherapy [9-11]. A better characterization of this proto-oncogene can lend insight to the pathogenesis and molecular mechanisms involved in the development of endometrial carcinoma.

We have preciously made nude mice transplanted with Ishikawa cells, which were stably transfected with HER2/neu plasmid and empty plasmid,respectively. The tumor volume and weight were measured.It showed that the tumor formation rate and tumor size in HER2/neu plasmid transfection group were significantly higher than those of the control group, which suggested that HER2 could promoted the growth of Ishikawa cells. In the present study, we confirmed that HER-2/neu mRNA and protein levels were significantly elevated in cells stably transfected with pcDNA3.1-HER2/neu compared with non-transfected cells or those transfected with empty vector. Using these cells, we identified the significant increases in the levels of COX-2 and P450arom. In addition, the E2 concentration was also significantly increased in cells stably transfected with pcDNA3.1-HER2/neu compared with non-transfected or empty vector-transfected groups. As an alternative approach, RNA interference technology was used for the down-regulation of HER2 expression in Ishikawa cells. The results showed that inhibition of HER2 in Ishikawa cells significantly induced the decrease of COX-2 and P450arom expression. Meanwhile, celecoxib, a selective COX-2 inhibitor, inhibited the expression of PGE2 and P450arom in the over-expressed HER2 Ishikawa cells. These results indicated that HER-2/neu induced the upregulation of COX-2, PGE2 and P450arom to promote the autocrine of E2 in endometrial carcinoma cells.

As a transmembrane glycoprotein, the cell membrane portion of HER-2/neu is the primary contributor to transduction of cell proliferation signals [12,13]. The tyrosine kinase activity of HER-2/neu is essential for COX-2 transcriptional activation [14] and regulates the expression of COX-2 via multiple pathways [15]. Over-expression of COX-2, which was detected in endometrial carcinoma, stimulated the proliferation and angiogenesis of cancer cell [16]. COX-2 also is an important rate-limiting enzyme in prostaglandin synthesis [13]. The endometrial prostaglandin E_2_ induced the activity of aromatase (P450arom) by up-regulating intracellular cAMP levels in endometrial stromal cells. COX-2 indirectly regulated the expression of P450arom by influencing the synthesis of PGE_2_[17]. P450arom is the rate-limiting enzyme catalyzing the final step in the conversion from androgen to estrogen. P450arom determined the levels of estrogen in normal and abnormal tissues directly, which maintained the estrogen-related physiologic functions and impacted the pathogenesis and prognosis of estrogen-dependent diseases [18]. High levels of HER-2/neu have been detected in endometrial carcinoma tissues and were found to correlate with tumor malignancy [19-21]. Our results suggested that HER-2/neu, as a potential upstream regulatory molecule in the COX-2/PGE_2_/P450arom signaling pathway, could play a critical role in estrogen-dependent endometrial carcinoma. These findings provided an improved understanding of the molecular mechanisms of estrogen-dependent endometrial carcinoma, and might instruct to screen the targets for hormone-dependent gynecologic tumors related to HER-2/neu.

## Competing interest

The authors declare that they have no competing interests.

## Authors’ contributions

XXM and SL, conception, experimental design and performance, data analysis and interpretation, manuscript writing; CW conception and design, data analysis and interpretation; LM, YQH and ZJY performed research; SL conception and design, financial support, provision of study material, final approval of manuscript. All the authors read and approved the final manuscript.
